# Controllable Fabrication of Sub-10 nm Graphene Nanopores via Helium Ion Microscopy and DNA Detection

**DOI:** 10.3390/bios14040158

**Published:** 2024-03-27

**Authors:** Zhishan Yuan, Yanbang Lin, Jieming Hu, Chengyong Wang

**Affiliations:** 1 School of Electro-Mechanical Engineering, Guangdong University of Technology, Guangzhou 510006, China; 2112101024@mail2.gdut.edu.cn (Y.L.); 2112201368@mail2.gdut.edu.cn (J.H.); cywang@gdut.edu.cn (C.W.); 2 Guangdong Provincial Key Laboratory of Minimally Invasive Surgical Instruments and Manufacturing Technology, Guangdong University of Technology, Guangzhou 510006, China; 3 State Key Laboratory for High Performance Tools, Guangdong University of Technology, Guangzhou 510006, China; 4 Smart Medical Innovation Technology Center, Guangdong University of Technology, Guangzhou 510006, China

**Keywords:** graphene nanopores, DNA detection, HIM

## Abstract

Solid-state nanopores have become a prominent tool in the field of single-molecule detection. Conventional solid-state nanopores are thick, which affects the spatial resolution of the detection results. Graphene is the thinnest 2D material and has the highest spatial detection resolution. In this study, a graphene membrane chip was fabricated by combining a MEMS process with a 2D material wet transfer process. Raman spectroscopy was used to assess the quality of graphene after the transfer. The mechanism behind the influence of the processing dose and residence time of the helium ion beam on the processed pore size was investigated. Subsequently, graphene nanopores with diameters less than 10 nm were fabricated via helium ion microscopy. DNA was detected using a 5.8 nm graphene nanopore chip, and the appearance of double-peak signals on the surface of 20 mer DNA was successfully detected. These results serve as a valuable reference for nanopore fabrication using 2D material for DNA analysis.

## 1. Introduction

Single-molecule detection technology has the advantages of high sensitivity, high resolution and high throughput in addition to being label-free, fast and low-cost [1]. This technology is widely used in the analysis of DNA [2], RNA [3] and proteins [4]. Moreover, nanopores are mainly categorised into biological and solid-state nanopores. Biological nanopores, such as α-haemolysin [5], phage phi29 [6] and MspA protein [7], are natural protein channels with good reproducibility that are low-cost and easily modified. Biological nanopores exhibit high detection resolution at the sub-nanometre scale [8] but have the disadvantages of unchangeable pore sizes, susceptibility to environmental influences and poor mechanical stability [9]. Alternatively, materials used as solid-state nanopores include Si_3_N_4_ [10,11], SiC [12], SiO_2_ [13,14], Al_2_O_3_ [15], graphene [16,17,18,19,20], MoS_2_ [21] and WS_2_ [22]. Solid-state nanopores, with their controllable shapes and sizes and good stability, are easier to integrate into biosensors for use in embedded structures and electronic devices. In the past 20 years, many researchers have used Si_3_N_4_ nanopores for biomolecular detection. However, the thinnest Si_3_N_4_ material still has a thickness of 2 nm [23], and at least six bases are located inside the nanopore sensor during the detection process, resulting in low detection accuracy [24] and difficulty in the differentiation of a single nucleotide. Ultra-thin two-dimensional (2D) materials, such as MoS_2_, WS_2_, transition metal dichalcogenides (TMDs) and graphene, have been investigated extensively. The single-layer thickness of MoS_2_ is 0.7 nm, and it can be used for long periods (i.e., hours or even days). The molybdenum-rich region around the borehole reduces DNA adhesion to it, enhancing its repeatability [25]. Garoli et al. [26] used FIB to successfully prepare nanopores with a pore size of less than 10 nm on MoS_2_ membranes and achieved high pattern repeatability to prepare multi-point nanopores. Monolayer WS_2_ has the same thickness as MoS_2_ [27], but its photoluminescence (PL) quantum yield is higher than that of MoS_2_, which makes the application of WS_2_ in optoelectronic devices possible [28]. Danda et al. [22] used TEM electron beams to drill nanopores with a sub-5 nm aperture on WS_2_ monolayers characterised by the PL spectrum and successfully identified various pore postures of double-stranded DNA. The van der Waals heterostructure of TMDs is a vertical stack of two 2D materials with strong light-harvesting capabilities [29]. Gu et al. [30] drilled nanopores with a pore size of less than 5 nm on the surface of the MoS_2_–graphene heterostructure by TEM and prolonged the dwell time of BSA translocation up to several hundred milliseconds. Compared with other 2D materials and TMDs, graphene is the thinnest 2D material, with a single atomic layer thickness comparable to nucleobase spacing [11]. In addition, graphene has excellent optical, chemical, electrical and mechanical properties, which can significantly improve the spatial resolution of biomolecule detection; thus, it has potential as a candidate material for nanopore sequencing. Although a weak interaction force is generated when the base comes into contact with graphene [31,32], inducing biomolecules to adsorb on the surface of graphene and causing structural changes or damage to graphene, which results in lower reproducibility, graphene is still a candidate material for nanopore sequencing due to its atomical thickness that can promote the detection precision of nanopore sensors greatly. Currently, the techniques applied in fabricating graphene nanopores include focused electron beam (FEB), focused ion beam (FIB), reactive ion etching (RIE), controlled breakdown (CBD) and helium ion beam (HIB) techniques [33]. Garaj et al. [19] fabricated graphene nanopores via FEB and achieved the detection of single DNA molecules. However, the FEB reorganises the atomic layer near the pore opening of graphene nanopores, leading to changes in its crystal structure and undesirable defects and damage, which increase the noise level [34]. Patterson et al. [35] fabricated nanopores in graphene membranes via FIB. However, the resolution of a FIB is limited by its beam spot diameter, beam spot shape and redeposition, making the fabrication of sub-10 nm nanopores difficult. Han et al. [36] used RIE to fabricate graphene nanopores with pore diameters of 1–50 nm; however, the efficiency and accuracy of the technique still need to be improved [37]. Rollings et al. [38] successfully fabricated nanopores in graphene using CBD; however, the random location of the generated nanopores is difficult to characterise, and additional complex methods are needed to assist in localisation [39]. Hall et al. [40] first fabricated nanopores in Si_3_N_4_ membranes via helium ion microscopy (HIM). The HIB technique has the advantages of a rapid fabrication process, high processing accuracy and high reproducibility, and it can handle multiple chips at the same time; thus, it can be used as a method for the large-scale fabrication of graphene chips at sub-10 nm scales [41].

In this study, HIM was utilised to fabricate graphene chips with sub-10 nm apertures, and the smallest graphene nanopore of 6 nm was fabricated. In addition, the detection of 20 base DNA was achieved on this basis, confirming the capability of graphene to improve spatial resolution.

## 2. Materials and Methods

### 2.1. Materials

KCl, EDTA, Tris and hydrochloric acid were purchased from Sigma-Aldrich (Shanghai, China). The 0.1 M KCl electrolytes were buffered with 10 mM Tris and 1 mM EDTA. Then, the pH of the electrolytes was adjusted to 8 with KOH, following some special instructions. DNA was provided by Jinsirui Biotechnology (Nanjing, China) and was synthesised from a single strand of DNA and two DNA probes. The DNA was diluted to 20 nM in 0.1 M KCl solution with 10 mM Tris and 1 mM EDTA. All solutions used were filtered and degassed before use.

### 2.2. Si_3_N_4_ Substrate Preparation

**Figure 1:**
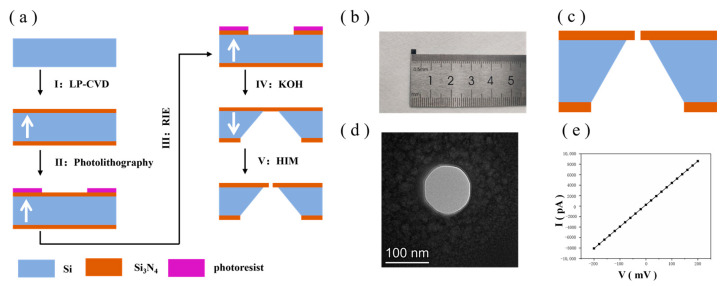
(**a**) Si_3_N_4_ substrate chip manufacturing. (**b**) Mobile phone photo (Huawei P50) of the Si_3_N_4_ chip. (**c**) Cross-section diagram of the Si_3_N_4_ chip. (**d**) TEM image of the Si_3_N_4_ nanopore. (**e**) I−V diagram of the Si_3_N_4_ nanopore in 1 M KCl buffer.

The process flow for the preparation of Si_3_N_4_ chips is shown in Figure 1a. The chip has a square shape with an overall size of 2.4 mm × 2.4 mm. Figure 1b shows a mobile phone photo of the Si_3_N_4_ chip. The specific steps are as follows: (I) A silicon wafer with a thickness of 200 µm was selected as a substrate with a crystallographic orientation plane of (1, 0, 0). Then, a layer of low-stress Si_3_N_4_ film with a thickness of 200 nm was deposited on the upper and lower surfaces of the silicon wafer substrate by the low-pressure chemical vapour deposition method using SiH_2_Cl_2_ and NH_3_ as the reaction gases. (II) One of the Si_3_N_4_ layer surfaces was coated with photoresist, which is defined as the backside of the chip (in the direction indicated by the white arrow), and a square photoresist window was patterned using photolithography. (III) Using RIE and employing CHF_3_, SiF_6_ and He^+^ as etching gases, Si_3_N_4_ in the square window unprotected by the photoresist was etched away, exposing the silicon substrate and removing the photoresist. (IV) The silicon substrate was etched via wet etching using a 30% KOH solution, heated in a water bath environment at 80 ℃ for 6 h and etched inward starting from the release window. Given that different crystal faces in the silicon crystal have different corrosion rates, the etching direction of the alkaline solution started from the exposed silicon window and etched downward along the (1, 1, 1) crystal face, eventually terminating after reaching the Si_3_N_4_ layer on the other side. As a result, a square Si_3_N_4_ self-supported membrane of 5 × 5 µm was obtained. (V) A 100 nm nanopore aperture was prepared on a silicon nitride substrate via HIM. Figure 1c shows the overall picture of the Si_3_N_4_ chip, Figure 1d shows the TEM image of the Si_3_N_4_ nanopore and Figure 1e shows the I−V diagram of the Si_3_N_4_ nanopore chip in 1 M KCl solution, which shows good linearity, indicating a good pore pattern. The entire Si_3_N_4_ substrate with nanopores serves as the take-up window for the layup of the graphene membrane in the subsequent steps.

### 2.3. Graphene Transfer and Characterisation 

**Figure 2:**
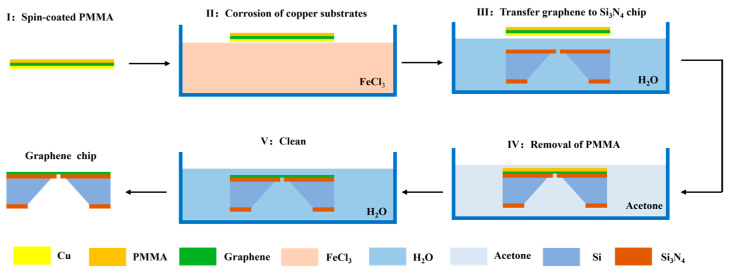
Flow chart of graphene membrane chip transfer process.

Graphene chips can be obtained by the wet transfer technique using the Si_3_N_4_ nanopore chip as the substrate. A single-layer graphene membrane was transferred to the top through the following experimental steps: (I) A piece of copper-based graphene material was provided, and a polymethylmethacrylate (PMMA) membrane was spin-coated on its graphene-covered side using a rotational speed of 2500 r/min and heated at 120 ℃ for 3 min to cure the PMMA membrane. (II) The copper-based graphene with the cured PMMA membrane was attached (copper substrate side down) to the surface of a 2 M FeCl_3_ solution and etched for 3 min. Then, a dropper was used to draw up the FeCl_3_ solution, and graphene was rinsed on the back of the copper foil 2–3 times. The copper substrate was left to completely dissolve for 8 h, after which the PMMA membrane and graphene flakes were floating on the surface of the FeCl_3_ solution. (III) To remove the FeCl_3_ solution from the backside of the graphene with the cured PMMA membrane, graphene with the cured PMMA membrane was fished from the FeCl_3_ solution using a clean slide and placed on the surface of deionised water to clean for 30 min. Graphene with the cured PMMA membrane was retrieved from the deionised water using a Si_3_N_4_ nanopore chip and annealed for 2 h at 85 °C to ensure that the graphene fit the target chip tightly. (IV) To completely remove the PMMA membrane from the surface of the graphene, the annealed chip was soaked in acetone three times. The first two soakings were performed for 30 min, and the third soaking was performed for 8 h. (V) To remove the acetone from the surface of the chip, the chip was placed in deionised water for 5 min and dried at 60 °C in an oven to obtain a graphene chip with a clean surface. A flowchart of the transfer process of the graphene membrane chip is shown in Figure 2.

**Figure 3:**
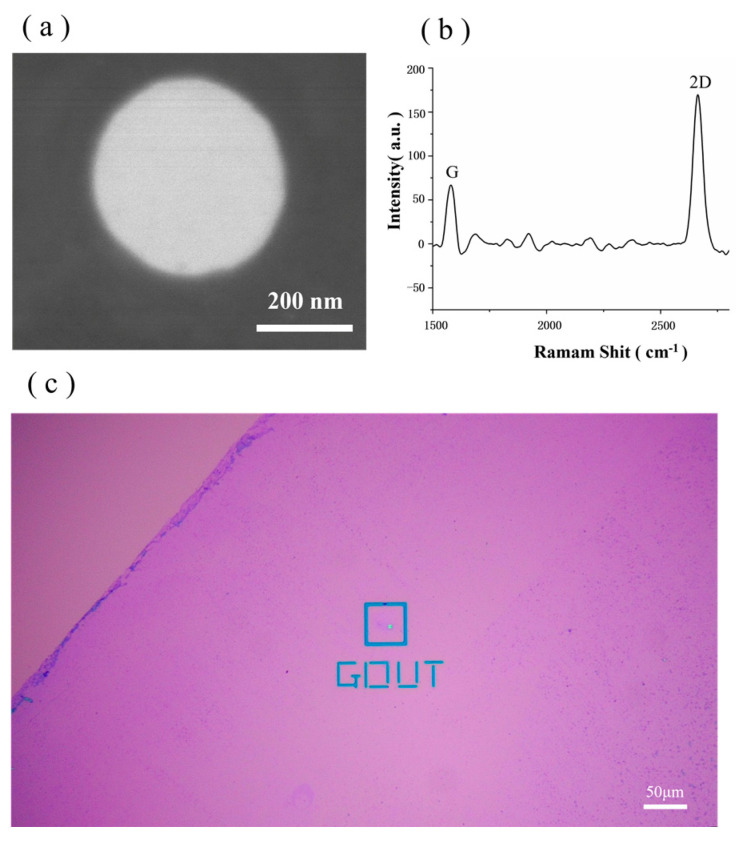
(**a**) Image of graphene chip captured via HIM. (**b**) Graphene Raman Shift acquired using a 532 nm band laser. (**c**) Optical microscope image of the chip after graphene transfer.

In this study, images obtained using HIM and Raman spectroscopy are used to characterise the graphene chip. As shown in Figure 3a, the HIM image shows that the graphene surface is complete, clean and bright. Furthermore, Raman spectroscopy is an ideal analytical tool for characterising graphene, and the structural features and properties of graphene, such as the number of layers, stacking mode and defects, can be determined by recording the Raman spectra of the material [42]. A 532 nm band laser was used to collect information on the number of layers in the graphene chip, and the results are shown in Figure 3b. In the figure, the G peak is roughly located at 1592 cm^−2^, and the shape of this peak is sharp. The 2D peak is roughly located at 2710 cm^−1^. The ratio of the G peak to the 2D peak is less than 1; thus, it can be determined that the graphene transferred to the Si_3_N_4_ substrate is in a single layer. Figure 3c shows an optical microscope image of the chip after the graphene transfer, and a clear difference between the graphene-covered area and the graphene-uncovered area is detected in the image (the triangular area in the upper left corner). It is clear to see that the larger graphene membrane covers on the membrane in the blue box in Figure 3c are where graphene nanopores will be created. In this experiment, the success rate for transferring graphene to the Si_3_N_4_ chip can reach 80% following the aforementioned steps.

### 2.4. Fabrication and Characterisation of Graphene Nanopores

The fabrication of graphene nanopores via HIM harnesses the energy transferred to the carbon atoms in graphene when they collide with high-energy ions, causing the carbon atoms to be sputtered out of the surface of the material, resulting in the formation of the nanopore [21]. The graphene nanopores were processed using the point processing mode of the HIM with a beam current of 1.5 pA and different processing doses and dwell times. The graphene nanopores were characterised via TEM. The point processing mode was selected because, in this mode, the beam spot acts on only one point, and the theoretically processed pore size is small for the area processing mode. The point processing mode was selected to maintain the beam current at a smaller value (1.5 pA) because, during the machining process, the graphene membrane at the window aperture must be imaged to locate the machining area, and a larger beam current will cause damage to the graphene, leading to the rupture of the graphene membrane. The graphene material is thin and does not require a large dose to achieve nanopore processing; thus, a small beam current will not affect the processing efficiency.

### 2.5. Characterisation of DNA Hybrid Strand Transport in Graphene Nanopores

**Figure 4:**
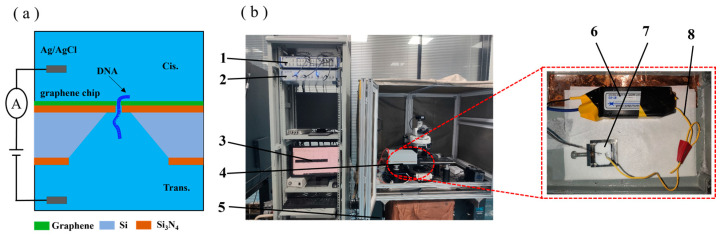
(**a**) Working principle diagram of the nanopore sensor. (**b**) Diaphragm clamp inspection device: 1, Axon digital-to-analogue converter 1550B; 2, Axon amplifier 700B; 3, computer; 4, Faraday shield box; 5, air float stage; 6, diaphragm clamp amplifier headstage; 7, flow cell; 8, Ag/AgCl electrodes.

A working principle diagram of the nanopore sensor is shown in Figure 4a. The 0.1 M KCl solution was added to the flow cell. The Ag/AgCl electrode was connected to a membrane clamp (Axon 700B, Molecular Devices, Shanghai, China). The inspection system of the diaphragm clamp is shown in Figure 4b. The voltage was applied to the trans side to conduct a blanking test with a sampling time of 2 min to ensure that the prepared graphene nanopores were free of contaminating signal sources. The 0.1 M KCl solution was removed from the cis side of the flow cell, and a 20 nM DNA solution was added. Then, a voltage of 200 mv was applied to the trans side, and the cis side was grounded. Finally, the signal of the DNA molecule passing through a nanopore was recorded. The experimental data were statistically analysed using Clampfit 10.7.0.3, Excel 2019 and Origin software 2018.

## 3. Results and Discussion

### 3.1. Influence of Processing Dose on Nanopore Size

**Figure 5:**
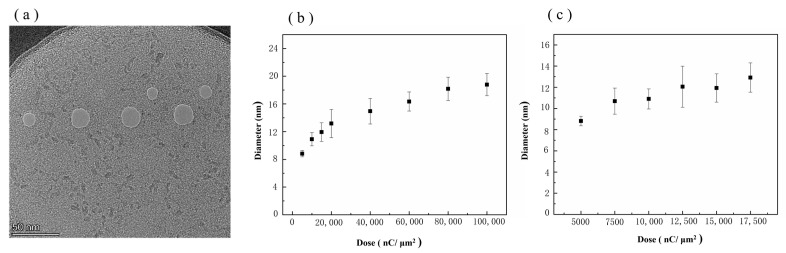
(**a**) TEM images of graphene nanopore chips processed via HIM at different ion doses. (**b**) Relationship between the size of the graphene nanopore and the ion dose in the range of 5000–100,000 nC/µm^2^. (**c**) Relationship between the size of the graphene nanopore and the ion dose in the low dose range of 5000–17,500 nC/µm^2^.

Ion beam processing is only related to the total delivered dose during processing, independent of the beam current; thus, the processing dose is used in this study to quantify the degree of processing. A diaphragm (20 µm) was used to control the ion beam during processing, and the processing beam current was −1.5 pA with a residence time of 2000 µs. Graphene nanopores with an average diameter range of 8.82–18.79 nm were obtained in the processing dose range of 5000–100,000 nC/µm^2^. Figure 5 shows the TEM image of a graphene nanopore fabricated via HIM at different doses. Figure 5a shows the relationship between the size of the graphene nanopore and the ion dose in the range of 5000–100,000 nC/µm^2^. The growth of the graphene nanopore undergoes two stages as the ion dose increases. In the low dose range of 5000–17,500 nC/µm^2^, the graphene pore size grows linearly with the increase in the ion dose, as shown in Figure 5c. However, with a further increase in the ion dose, the growth of the nanopore size slows down in the range of 17,500–100,000 nC/µm^2^.

**Figure 6:**
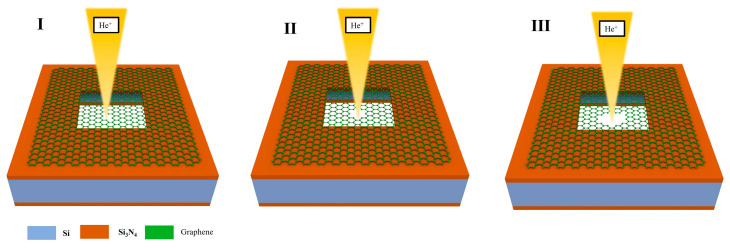
Ion dose and graphene nanopore etching model.

This phenomenon can be explained by the model of graphene nanopores etched using helium ions shown in Figure 6. The process of generating nanopores through the etching of graphene using helium ions can be divided into three stages. In the first stage, the defects in graphene created by the action of the helium ions will interact with the deposition of hydrocarbon contaminants from the HIB, resulting in the healing and reweaving of the defects [19]. Therefore, helium ions have a negligible effect on graphene at low doses. However, as the etching dose increases, Frenkel and Stone–Wales defects are created in the graphene. Frenkel defects are caused by the carbon atoms colliding with the helium ions, resulting in the displacement of the carbon atoms from their original region, forming a pair of vacancies and interstitial atoms. In the Stone–Wales defects, the C–C bonds of graphene atoms are exposed to helium ions and rotated by 90°, which results in the rearrangement of the four adjacent hexagonal carbon lattices into two pentagonal and two heptagonal carbon lattices [43]. At this stage, mainly localised defects are produced.

In the second stage, with the gradual increase in the ion dose, a large number of defects will absorb each other and form a nanopore, the pore size of which increases linearly with the ion dose [44]. The linear relationship between ion dose and nanopore size shown in Figure 5c also proves the existence of this process. Given the Gaussian distribution of the HIB energy [45], ion beam centres with high intensities are involved in the etching process during the low-dose phase of the etching process. In the third stage, as the dose increases and the nanopore aperture expands, the edge of the nanopore will gradually move outward from the centre to the edge of the ion beam. The high-energy part at the centre of the HIB will pass through the nanopore, whereas the low-intensity part at the edge of the HIB continues to participate in the etching of graphene nanopores [45]. Therefore, in the process of graphene nanopore fabrication using the HIB, more stable and controllable fabrication of graphene nanopores can be achieved by controlling the processing dose so that the etching process takes place in the second stage [46].

### 3.2. Effect of Residence Time on Graphene Pore Size

The process control programme of HIM ensures that the number of times the HIB is applied to the sample during processing can be adjusted by setting the ‘repeat times’ or ‘dwell times’. The set total processing dose is divided into different deliveries, and the dwell time refers to the amount of time the HIB is applied to the sample during each delivery. The relationship between the total processed dose (*T*), the number of repeat times (*n*) and the dwell time (*t*) satisfies Equation (1):

*T* = *n* × *t* (1)

**Figure 7:**
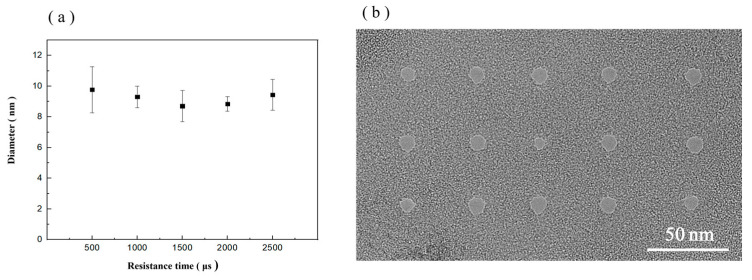
(**a**) Relationship between pore size and different dwell times. (**b**) TEM images of graphene nanopores fabricated via HIM at different dwell times.

The longer the dwell time is, the more concentrated the dose in a single delivery. The shorter the residence time is, the more dispersed the dose in a single delivery. However, this change does not affect the total delivered dose. In this study, the effects of different dwell times on graphene nanopore processing were investigated. Graphene nanopore processing was conducted using dwell times of 500, 1000, 1500, 2000 and 2500 µs with a diaphragm of 20 µm, an HIB current of 1.5 pA and a dose of 5000 nC/µm^2^. The processing results are shown in Figure 7a. The average pore diameters of the nanopores processed using a dwell time of 500–2500 µs are 9.77, 9.31, 8.07, 8.84 and 9.43 nm. The relationship between pore size and different dwell times is shown in Figure 7a, and the TEM images of the nanopores fabricated via HIM at different dwell times are shown in Figure 7b. The diameters of the processed nanopores vary within 1.7 nm, and no significant relationship between graphene nanopore diameter and dwell time is observed. Although the residence time varied, it did not meaningfully affect the total dose of helium ions acting on the graphene.

### 3.3. Fabrication of Sub-10 nm Graphene Nanopores 

**Figure 8:**
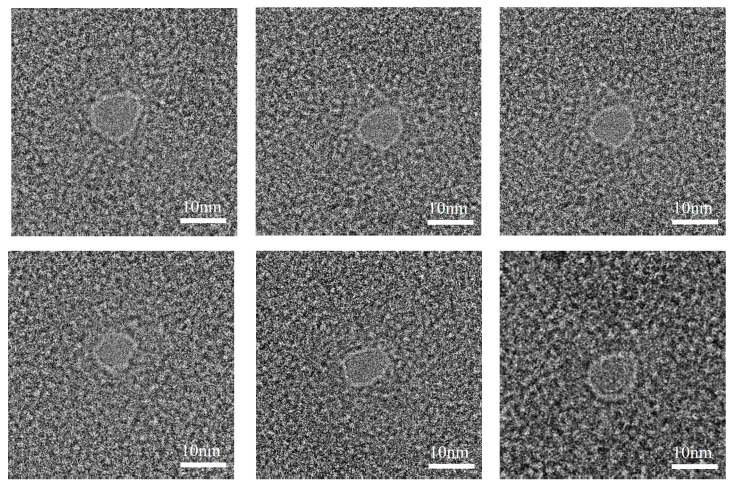
TEM images of graphene nanopores with an average pore size less than 10 nm.

A batch of graphene chips was processed using HIM under the following conditions: a diaphragm of 20 µm, a processing beam current of 1.5 pA, a processing dose of 5000 nC/µm^2^ and a dwell time of 2000 µs. Figure 8 shows the TEM images of graphene nanopores fabricated via HIM, where the diameters of the graphene nanopores are less than 10 nm. Notably, the processing of graphene membranes with these parameters can maintain the diameter of the graphene nanopore to less than 10 nm. Because the graphene membrane is thin, a large total dose is not required to etch out the nanopore. The graphene membrane will not be damaged under the small beam current of 1.5 pA, even if the HIM images the processed area for a long time. Using a processing dose of 5000 nC/µm^2^, a more stable and controlled graphene nanopore fabrication can be achieved by controlling the processing dose such that the etching process remains in the second stage during etching, in which there is a high-intensity ion beam centre. The successful preparation of sub-10 nm graphene chips can lay the foundation for subsequent DNA detection experiments.

### 3.4. The Transport Characteristics of DNA in Graphene Nanopores

**Figure 9:**
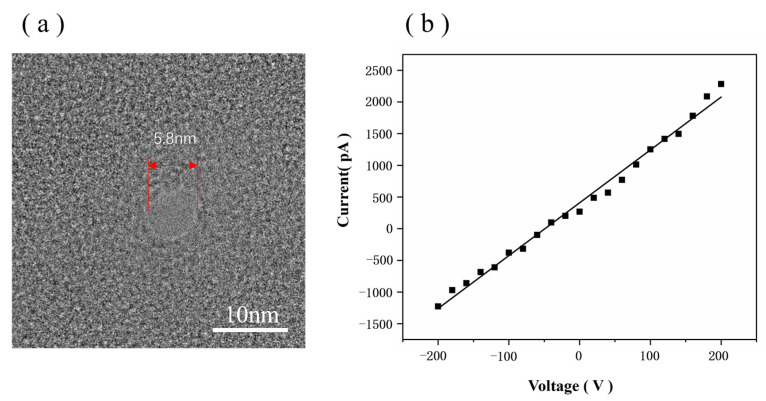
(**a**) TEM image of the graphene nanopore chip used for detection. (**b**) IV plots of graphene nanopore.

To validate the functionality of our HIM-fabricated nanopores, we detected DNA using graphene nanopores in 0.1 M KCl. The TEM image of the graphene nanopore used for detection is shown in Figure 9a; its nanopore diameter is 5.8 nm.

The effective diameter of the graphene nanopore can be calculated from the ionic conductance of the pore, *G*, based on Equation (2) [47]:

(2) $$G=\sigma {\left[\frac{4l}{\pi d^{2}}+\frac{1}{d}\right]}^{-1}$$

where *σ* is the bulk electrolytic conductivity (for 0.1 M KCl, *σ* = 1.28 S/m), *d* is the diameter of the nanopore, and *l* is the thickness of the nanopore membrane. Given that the thickness of graphene is only 0.335 nm, the 4*l*/*πd*^2^ part in Equation (2) can be neglected [48]. Thus, the current formula for the graphene nanopore can be expressed as follows:

G = σd(3)

**Figure 10:**
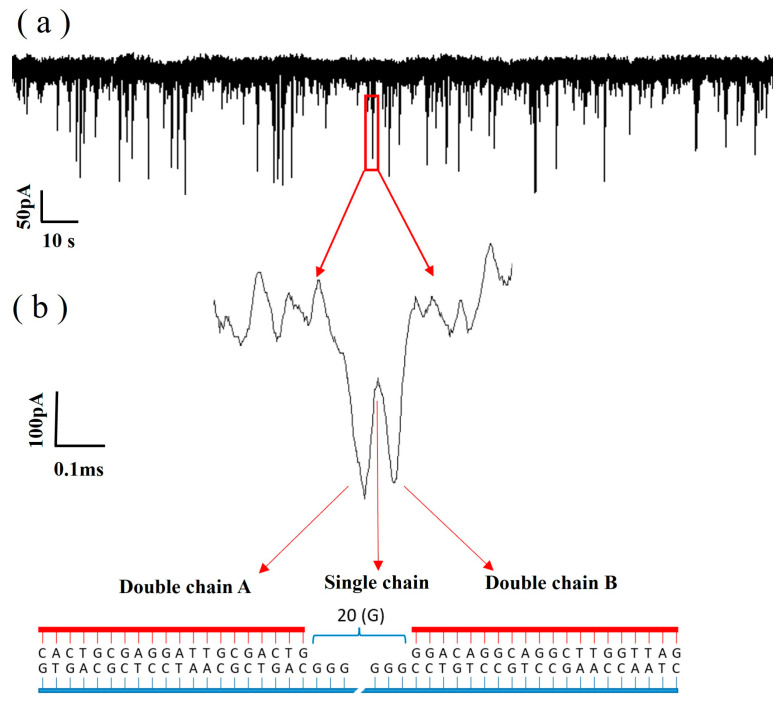
(**a**) Ionic current trace of the translocation of DNA through a 5.8 nm nanopore in 0.1 M KCl with an applied voltage of 200 mV. (**b**) The bimodal signal is an amplification of one of the signals.

Using Equation (3), the diameter of the graphene nanopore was determined to be 5.8 nm, which is suitable for use in DNA molecule detection. The ionic current trace at a 100 mV bias voltage is shown in Figure 10a, in which the pulse signals indicate the events corresponding to DNA translocation through the pore from the cis chamber to the trans chamber. Figure 10b shows one of the signals in the ionic current trace, which is a double-peaked signal and is related to the structure of the DNA. Figure 10b shows the structure of the DNA, which is synthesised from an ssDNA strand with two probes; thus, the structure is ‘dsDNA A–ssDNA–dsDNA B’. When dsDNA A passes through the graphene nanopore, the blockage of the double strand increases the signal drop. When the ssDNA region passes through the graphene nanopore, only a volume equivalent to half of the diameter of the dsDNA blocks the ions in the nanopore, resulting in a decrease in the signal drop. When dsDNA B passes through the graphene nanopore, the same effect as that of dsDNA A can be observed; thus, the double-peak signal appears, as shown in Figure 10b. Therefore, the appearance of the double-peak signal confirms that the ‘dsDNA A–ssDNA–dsDNA B’ structure successfully passed through the graphene nanopore.

**Figure 11:**
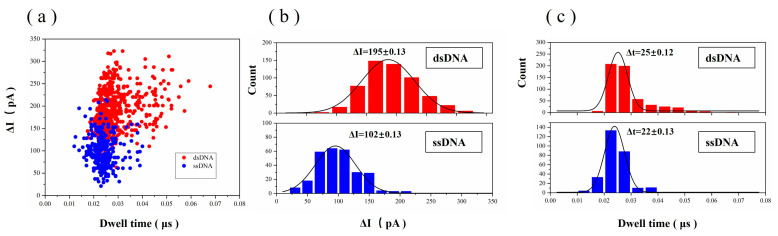
(**a**) Scatter plot of dwell time versus current blockade at 200 mV for ‘dsDNA A–ssDNA–dsDNA B’ translocation through the graphene nanopore. The bimodal signal was divided into two statistical parts. The two peaks towards the bottom are dsDNA passing through the graphene nanopore, and the middle peak is ssDNA passing through the graphene nanopore. (**b**) Histograms of ∆*I* for ‘dsDNA A–ssDNA–dsDNA B’ translocation through the graphene nanopore fitted with a Gaussian distribution. (**c**) Histograms of dwell time for ‘dsDNA A–ssDNA–dsDNA B’ translocation through the graphene nanopore fitted with a Gaussian distribution.

A total of 280 bimodal signals were selected and counted, and three peaks of these bimodal signals were counted separately. The two downward peaks represent dsDNA passing through the graphene nanopores, and the upward middle peak represents ssDNA passing through the graphene nanopores. Figure 11a presents a scatter plot of translocation dwell time versus current blockade, ∆*I*, for ssDNA and dsDNA passing through the graphene nanopores. Figure 11b shows the frequency statistics of the blocking current. The blocking current of dsDNA is mainly distributed in the 160–220 pA range, showing certain Gaussian curve characteristics, and the peak Gaussian value is approximately 195 pA. The blocking current of ssDNA is mainly distributed in the 80–140 pA range, and the peak Gaussian value is approximately 102 pA. Figure 11c shows the frequency statistics of the dwell time. The dwell time of dsDNA is mainly distributed in the 20–35 µs range, and the Gaussian peak value is approximately 25 µs. The dwell time of ssDNA is distributed in the 15–30 µs range, and the Gaussian peak value is approximately 22 µs. The DNA hybridisation strand translocates at a rate of 0.7–0.8 µs/base in a 5.8 nm graphene nanopore.

The blocking current from DNA translocation is related to the reference current as follows [49]:

(4) $$\frac{∆I}{I_{0}}=\frac{d_{DNA}^{2}}{d^{2}}$$

where ∆*I* is the blocking current, *I*_0_ is the reference current, *d_DNA_* is the diameter of the DNA molecule and *d* is the diameter of the graphene nanopore. For dsDNA, substituting ∆*I* = 195 pA, *I*_0_ = 1460 pA and the graphene nanopore size *d* = 5.8 nm into Equation (4), the actual diameter of dsDNA was calculated to be 2.119 nm, which is similar to that of the theoretical diameter of dsDNA *d_DNA_* = 2.12 nm [50]. For ssDNA, substituting ∆*I* = 102 pA, *I*_0_ = 1460 pA and the graphene nanopore size *d* = 5.8 nm into Equation (4), the actual diameter of ssDNA was calculated to be 1.533 nm, which is slightly larger than the theoretical diameter of ssDNA *d_DNA_* = 1.29 nm [50] because the single-stranded region of the hybridised strand of DNA is different from the ssDNA strand alone. When the single-stranded region of ‘dsDNA A–ssDNA–dsDNA B’ passes through the graphene nanopore, influenced by the access resistance, the double-stranded region of ‘dsDNA A–ssDNA–dsDNA B’ affects the spatial occupation, resulting in the discharge of more ions, and the blocking current increases.

## 4. Conclusions

In this study, the preparation of graphene nanopores with a sub-10 nm pore size was explored based on HIM to determine the mechanism behind the effect of the processing dose and residence time of the HIB on the processed pore size. The diameters of the fabricated graphene nanopores were statistically analysed under different dose conditions. Notably, the growth of the graphene pore diameters underwent two stages with the increase in dose: first, linear growth, and second, a slowed-down growth rate. The principle of helium ion etching in graphene nanopores was analysed, and it was determined that the processing dose should be controlled so that the etching process occurs in the second stage, as it results in a more stable and controllable fabrication of graphene nanopores. By statistically analysing the diameters of the fabricated graphene nanopores with different residence times, the residence time was determined to have only a slight effect on the fabrication of graphene nanopores. Finally, batch fabrication of sub-10 nm graphene nanopore chips was performed using helium ions, and the smallest nanopore diameter obtained using this processing technique was 5.8 nm, demonstrating the feasibility of the given parameters. The capability of the sub-10 nm graphene nanopore chip to detect DNA was investigated, and the detection of ultrasensitive signals from 20-base ssDNA was achieved. The results of this study can help improve the sensitivity of nanopore detection of DNA molecules and thus enhance the development of solid-state nanopores in DNA molecular sequencing technology.

## Data Availability

Data are contained within the article.
